# Understanding Motor Skill Learning as Related to Dentistry

**DOI:** 10.3390/dj9060068

**Published:** 2021-06-11

**Authors:** Mohamed El-Kishawi, Khaled Khalaf, Tracey Winning

**Affiliations:** 1 Preventive and Restorative Dentistry Department, College of Dental Medicine, University of Sharjah, Sharjah P.O. BOX 27272, United Arab Emirates; kkhalaf@sharjah.ac.ae; 2 School of Dentistry, the University of Adelaide, Adelaide, SA 5000, Australia; tracey.winning@adelaide.edu.au

**Keywords:** motor skills, learning theories, dentistry, self-consciousness, working memory

## Abstract

Learning dental procedures is a complex task involving the development of fine motor skills. The reported use of theories and/or evidence for designing learning activities to develop the fine motor skills needed for dental practice is limited. The aim of this review is to explore the available body of knowledge related to learning motor skills relevant to dentistry. Evidence from studies investigating motor skill learning highlights the negative impact of self-focus and self-regulation on learning outcomes, particularly during the early stages of learning. The development of activities and schedules that enable novices to demonstrate characteristics similar to experts, without the reported long period of ‘deliberate practice’, is clearly of value. Outcomes of learning implicitly are important in dentistry because working under stressful conditions is common, either during undergraduate study or in practice. It is suggested that learning implicitly in the simulation stage can reduce disrupted performance when transitioning to clinical settings. Therefore, further investigation of effective methods for learning dental fine motor skills is indicated, using approaches that result in robust performance, even under stressful conditions.

## 1. Introduction

Procedural and cognitive skills are essential abilities for clinical dental practice. Students learn these skills during simulated clinical activities designed to ensure that they achieve a satisfactory level prior to proceeding to direct patient care. These simulated activities have associated high costs in terms of staffing and facilities [1,2,3]. To optimize learning in these settings, the design of relevant learning activities needs to be informed by theory and based on evidence.

However, there has only been limited research conducted in relation to the design of the most effective and efficient methods for learning the complex cognitive and fine motor skills required for patient care in dentistry [1,4,5,6]. Similarly, there are few publications discussing the rationale and design of simulation and clinical endodontic learning activities [7,8,9].

Other than investigation of the design and diameter of hand file handles and the effect of the fit of gloves on performance [10,11,12], there has been limited use of learning theories to explicitly inform the design of simulation and clinical dental learning activities [6,13,14,15]. The purpose of this review is to explore the available body of knowledge related to learning motor skills in dentistry. The review also aims to familiarize the reader with definitions and theoretical explanations concerning motor skill learning based on extensive research. Please see Table 1 below describing terms used in this article.

## 2. Materials and Methods

The following electronic databases were searched to identify relevant articles to our topic: Google scholar, Scopus, PubMed, and Medline until October 2020. The search was carried out by two investigators to eliminate any potential bias in selecting the relevant articles. The following keywords were used to conduct a comprehensive search so that no key studies were missed during the search: motor skills, learning theories, dentistry, working memory, and self-consciousness, as well as, MeSH terms i.e., “dentistry”, “motor skills”, “learning” were used to conduct our comprehensive search. Inclusion criteria: all types of studies investigating motor skill learning as related to dentistry and discussing definitions and theoretical concepts concerning motor skill learning.

## 3. Motor Skill Learning Theories

Fine motor skill learning requires the control and integration of a range of stimuli and responses to be able to perform the desired motor task. How can we explain, support, or predict how people learn these skills? Several learning theories have been developed to explain how learning motor skills occurs and what stimulates individuals to learn and change. In dentistry, understanding relevant learning theories is essential for dental educators to be able to design effective learning activities, with a clear rationale that supports their dental students’ learning. Below, we will discuss five key theories that have relevance to learning procedural skills and declarative knowledge in dentistry. Specifically, these are Schema theory, Cognitive Load theory, OPTIMAL theory of motor learning, the Novice-Expert continuum and deliberate practice principles, and Reinvestment theory (Table 2).

### 3.1. Schema Theory

Theories of motor skill acquisition were initially conceptualized by behavioral psychologists based on the associations between stimuli and responses [21]. The role of cognition in motor skill acquisition was first emphasized by Adams [22]. Adams postulated that motor skill learning included a combination of motor behavior with a variety of cognitive processes in addition to the development of strategies that can be used to execute a motor task.

The way in which feedback and error detection affect learning was a fundamental element of Adams’ [22] theory of motor control. According to this theory, learners usually hold a reference of accuracy that determines a desired outcome of the movement and a feedback process that perceives error between the learner’s desired movement and the actual movement produced [22]. Research findings suggested that Adams’ views were true for movements that are relatively slow [16]. Slow movements provided learners with a chance to evaluate their performance and to detect any error between the desired movement and the actual movement by way of a feedback mechanism. Adams’ theory has several limitations [16]. It does not explain how rapid movements are learned and controlled. To achieve rapid movements, a motor plan needs to be structured in advance, which does not allow for feedback during the movement. Another reported limitation is the effect of random practice on the accuracy of the perceptual recall of movement stored in the central nervous system [23]. Despite these limitations, Adams’ theory represented a step forward in understanding motor skill learning and paved the way for newer theories.

Schema theory, first proposed by Schmidt [16], suggested that a motor program (i.e., stored muscle commands) contains general rules that may be applied to different environmental or situational contexts through the contribution of an open-loop control process and generalized motor programs (GMP). Schema contain the common rules that generate the spatial, temporal muscle behavior designed to achieve a specified movement [24]. Therefore, when learning new movements, a person may produce a new GMP based on the choice of parameters (e.g., to reduce issues with the novel movement), or improve an existing GMP (which helps minimize the storage problem of multiple GMP), depending on previous experience with the movement and task context.

**Figure 1:**
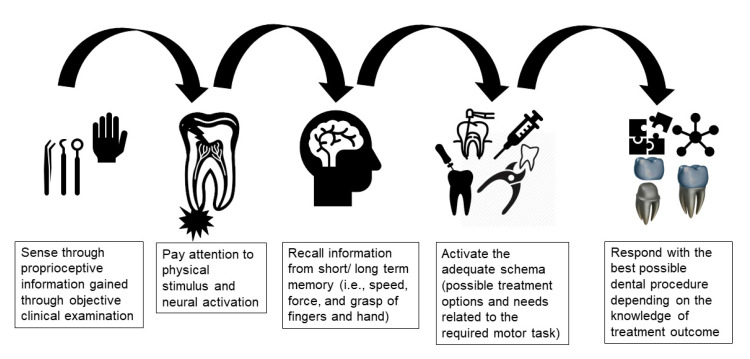
Schema theory as relevant to dentistry.

Schema theory proposed that after generation of a movement, four components are usually stored in memory: (a) the initial conditions (i.e., the proprioceptive information of the limbs and body); (b) the response specifications for the motor program, which are the parameters used in the generalized motor program (e.g., speed and force); (c) the sensory consequences of the response produced, which consist of information about how the movement felt, looked and sounded; and (d) the outcome of that movement with knowledge of the results [16]. Schema theory proposes that motor learning involves ongoing processes that update the recall and recognition schemas with every movement that is performed [24]. For example, initial motor movements related to a dental procedure (e.g., cavity preparation) are usually stored from proprioceptive information gathered from clinical examination. This would activate a memory recall of previous clinical experience related to hand/fingers speed and forces required to achieve the motor task (i.e., caries excavation). The result of this movement would depend on the stored knowledge about the desired procedure outcome (i.e., cavity outline form) of this dental procedure (Figure 1). Despite its deficiencies, schema theory has triggered the development of alternative ideas and provided a model from which new theoretical positions have been proposed. Schema theory is the most commonly used theory, either explicitly or implied that has been used to explain procedural skill development in dentistry [25].

However, for many scholars, including Schmidt himself, Schema theory no longer provides a satisfactory theoretical basis for understanding motor skill learning. In particular, more recent findings cast doubt on the cognitive-based assumptions of Schema theory, and it can only provide incomplete explanations of how motor skills are acquired [23,26]. For example, this theory is unable to describe how people are capable of learning through observation in the absence of cutaneous sensory feedback or movement [27].

Another issue related to Schema theory is the theoretical role of augmented feedback, when retention and transfer tests are conducted [28]. Feedback about motor movement can be divided into inherent (intrinsic) feedback, and augmented (extrinsic) feedback [28]. Specifically, inherent feedback is related to information about a motor task gained by the performer through various sensory channels during or after the execution of a motor movement, depending on the nature of the task. Augmented feedback, on the other hand, is related to information provided about a movement task that is supplementary to, or that reinforces, the inherent feedback [28]. Studies conducted by Lai and Shea [29,30]. showed that manipulation of feedback about performance results under different practice conditions did not result in consistent improvement in skill. Specifically, reducing the frequency of results feedback, achieved better performance stability and enhanced the learning of the motor tasks during random practice using a mixture of variable motor tasks during a practice session (i.e., ABC, BCA, CBA). In contrast, repetition of a single task during a practice session (block practice; i.e., AAA, BBB, CCC) resulted in an improvement of the motor task learning [15]. Therefore, Schema theory is not able to explain the effect related to manipulations of frequency of feedback on performance outcomes on motor task variables. In addition, this theory is limited in regards to the kind of characteristics in a motor program that are affected by manipulations of feedback about results of performance (e.g., timing, pattern and movement sequence), and the influence of variability and order of practice on the acquisition of the motor skills [26].

### 3.2. The OPTIMAL Theory of Motor Learning

The OPTIMAL (Optimizing Performance Through Intrinsic Motivation and Attention for Learning) theory considers the social and cognitive nature of motor behavior [17]. The application of the OPTIMAL theory for enhancing motor performance and learning in clinical settings involves discovering the correct approaches to support motivation and directing attention to the desired outcome of the fine motor task. In a typical dental training setting, the clinical instructor decides on the task to be practiced, describes how to perform the movements of the task, provides corrective instructions and feedback that relate to the orientation of the hand/finger’s movements. These instructions often include descriptions of the movements of a particular part(s) of the hand or fingers in relation to other body parts [31]. Instructions focusing on specific body movements, is described as having an ‘internal focus’. On the other hand, instructions that direct a learner’s attention to the effect of the movement are described as having an ‘external focus’ [32]. Literature on attentional focus effects have shown that slight modifications in the provided instructions can have a major effect on learning and performance [31]. When applying this concept in endodontics, it appears that providing instructions characterized by an external focus of attention (e.g., cleaning and shaping root canal space, and advancing the instrument) has more learning advantages, contrary to an internal focus of attention (e.g., angulation of the hand instrument inside the canal, movement or grasp on a hand instrument).

### 3.3. Cognitive Load Theory

Many contemporary theories of motor skill learning identified the importance of cognitive processes during motor skill acquisition, particularly in the initial stages of learning [33,34]. The initial stage of motor skill learning (i.e., cognitive stage/declarative stage) involves cognitive processing of verbal/visual instructions related to the task and rehearsal of the task in working memory. This cognitive processing facilitates the interpretation of the instructions required to perform the task [35]. Cognitive load theory (CLT) is related to working memory characteristics and instructional design [36]. It was developed to provide guidelines to assist in presenting instructional material in a way that facilitates learning motor activities and optimizes performance [37]. The history of this theory goes back to the 1950s when Miller [21] first described the limited nature of working memory and noted that humans are only able to hold seven, plus or minus two, pieces of information in their short-term memory. Subsequently, Sweller [18] further developed cognitive load theory to inform instructional design principles and strategies supported by a model of human cognitive architecture.

CLT is based on the assumption that the human cognitive system is limited by the fact that working memory (i.e., short-term memory) can only store and process a small amount of information for a few seconds [38]. This limitation in working memory capacity and duration is restricted to new information retrieved through sensory memory [39]. However, if information is obtained from long-term memory, these limitations do not exist. As reviewed by Sweller et al. [39], long-term memory is believed to store information as cognitive schemas, which may vary in complexity and automation. Expertise in humans is achieved by knowledge built up by these schemas. Careful and gradual combining of simple ideas to become more complex can result in the development of expertise in novice learners. The organization of knowledge by schemas can extensively reduce working memory load as highly complex schemas can be processed as a single element in working memory [40].

Limitation in the capacity of memory arises when handling completely new and unorganized information [41]. This limitation might be related to the increased number of elements to be organized, which increases the number of possible combinations of elements required to be tested during any problem solving process [40]. This memory overload problem can be compensated for by limiting the number of information units that are processed at the same time. This can be achieved by organizing information in long-term memory using schema construction processes, therefore reducing the extraneous cognitive load [40]. During problem solving processes, schemas can be built up by putting elements together (i.e., chunking), and/or combining new elements with already existing schemas in long-term memory [42]. Schemas can then be handled as a single element in working memory, which can significantly reduce cognitive load related to the performance of future tasks. Properly designed instructions should support schema construction, as well as encourage schema automation, which can help free working memory capacity for other activities.

The load on working memory may be influenced by the intrinsic environment of the learning tasks (*intrinsic load*), by the way tasks are presented (*extraneous load*), and by the actual learning that occurs (*germane load*) when handling intrinsic load [40].

Based on cognitive load theory, both intrinsic and extraneous cognitive loads are added and related when learners are presented with a task [40]. If intrinsic load is low, a high extraneous load resulting from poor instructional design might not be detrimental to learning as the total cognitive load is within the limits of working memory. When teaching complicated tasks (e.g., root canal preparation) involving greater interaction between elements involved in a task (i.e., motion, sequence, force, and tactile sensation in root canal preparation), the combined intrinsic and extraneous loads are likely to exceed working memory capacity and result in overload. The more that extraneous cognitive load is reduced, the more working memory resources can be dedicated to intrinsic cognitive load, which enables easier induction of a germane cognitive load for learning [40].

Cognitive Load theory has had a major impact on educational research and instructional design [43]. However, some critical questions have been raised concerning its conceptual clarity, validity of instruments used to measure cognitive load, and generalizability of its outcomes in different contexts and populations [36]. For example, there remains a lack of clear distinction between intrinsic, extraneous, and germane cognitive load. Moreover, measurement of cognitive load using self-reported questionnaires is often presented with no standard format and with differences in the number of items used for the survey. However, these measures cannot be used to measure cognitive overload (i.e., when working memory capacity is exceeded) [36].

### 3.4. Novice-Expert Continuum and Deliberate Practice Principles

**Figure 2:**
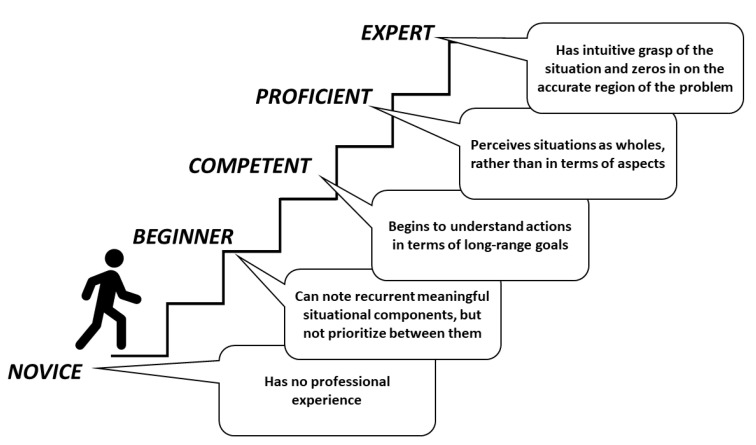
Pedagogical Needs and Limitations.

To develop expert understanding and skillful performance, Dreyfus et al., [19] suggested a five-stage development continuum beginning with novice level, moving through advanced beginner, to competent, proficient, and expert (Figure 2). A learner in training for a professional role develops from a true novice (a beginner) through a sequence of stages where capacities are gradually improved by trial-and-error learning and continual approximation supported by appropriate supervision. Dreyfus and colleagues [19] identified that the safe practitioner stage (competent) is a stage where the learner can perform the basic tasks related to a professional role and resolve common problems without assistance. This stage is the starting point for obtaining smooth, consistent, and accurate performance that is characteristic of true expertise [33].

The development of a graduate from a professional education program to become competent, proficient or even hold some aspects of expertise, depends on many factors [33]. These factors include the difficulty of the skills to be acquired, practice frequency, prospects for gradually increasing levels of challenge and responsibility for the task, and mentor availability to act as an instructor and role model [44]. Dental school graduates will generally not have the ability to perform as experts immediately after graduation, but hopefully can perform at a competent level for the essential skills associated with general dentistry [33]. With further practice and progress, it is anticipated that they will become experts.

Differences between experts and novices are often related to how they structure, analyze, and use information [45]. Expert practitioners have integrated neural networks that enable instant recovery of information related to task performance or assessment of a problem [38]. Novice learners, on the other hand, find it difficult to bring together isolated pieces of information. Novices use an ineffective trial-and-error method because of the deficiency in their pre-existing networks [33]. Students may possess some information (i.e., from textbooks or manuals), but the information is isolated and often not related to other topics. To develop problem solving ability, students need to convert their disorganized acquired information (i.e., pieces of data) from textbooks and lectures into connected chains of networked knowledge, which have meaning, significance, and recognized value that can be described in an individual’s own words [46].

Research studies have highlighted that novices can benefit from learning motor skills with minimal conscious involvement as performance is maintained when high levels of cognitive effort are required [47]. Furthermore, it is evident that novices who learn without attending to and monitoring their movements demonstrate characteristics that are similar to the performance of professionals with comprehensive knowledge and skills (i.e., experts). The development of activities and schedules that enable novices to demonstrate characteristics similar to experts, without the reported long period of ‘deliberate practice’, is clearly of value [45].

### 3.5. Reinvestment Theory

The general distinction between conscious and non-conscious features of motor learning processes is considered a starting point to explain a variety of motor learning models [48]. Research into motor skill acquisition has demonstrated that motor skill learning is often disrupted by distraction and self-focus, especially under stressful conditions [49,50]. When distracted, the attention of the performer becomes focused on stimuli that are not related to the motor task. Self-focus, on the other hand, can direct attention in a way that involves self-regulation and self-evaluation in an attempt to match the required standard of performance [51]. Masters (1992) grouped the range of views of self-focus control behaviors under the term ‘reinvestment’ [51,52].

The theory of reinvestment relates to the conscious attempts by performers to ensure the quality of their performance, by observing (i.e., movement self-consciousness) and controlling (i.e., conscious motor processing) their own movements using explicit processes involving working memory [51]. As a result, of this observation and control, disruption of automated execution of some motor skill components can occur, which results in poor movement quality, and subsequent breakdown of performance [20].

The difficulty of the task also influences the level of disruption from reinvestment. Propensity to reinvest has been suggested to be associated with more complex tasks rather than simple tasks [53]. This is relevant to learning how to prepare root canals or access cavity, as these are complex tasks involving different procedures that require cognitive access to both procedural knowledge (related to stages of treatment, sequence of instruments and materials, and different mechanical techniques used) and declarative knowledge (related to the anatomy of the root canal system, diagnosis of the case, and choice of the most suitable material and instrument in relation to the tooth/patient condition). Therefore, the impact of reinvestment during complex dental procedures might be more disruptive for performance.

#### Implicit and Explicit Learning

The negative effect of reinvestment can potentially be prevented by emotion control training, training performers to avoid conscious control of their behaviour, distraction techniques, or directing performers to an external focus of attention [51]. Another possible way to prevent reinvestment is by using implicit methods for learning motor skills. Implicit learning of motor skills includes learning skills without the accumulation of conscious verbal knowledge (e.g., rules) about motor task performance, such that these “implicitly learned skills are (unconsciously) retrieved from implicit memory” [48]. Therefore, by learning implicitly, the aim is to limit the accumulation of movement-specific knowledge, decrease dependence on declarative knowledge structures during motor task performance, and minimize testing of hypotheses related to movements that are aimed at improving performance [54]. Studies have found that the value of implicit motor learning in novices exceeds the expected objective of acquiring motor skills by also showing robust performance under conditions of stress conditions, fatigue and when high levels of cognitive effort are required (e.g., when performing an additional or secondary task) [55].

An implicit approach contrasts with the conventional approach to learning motor skills in dentistry and surgery, namely learning explicitly by consciously following detailed textual, visual, and/or verbal instructions related to carrying out a motor skill task [56]. An explicit learning process consists of cognitive (declarative) stages and depends on involvement of working memory [48]. Research on motor skill learning during laparoscopy procedures has indicated that using explicit learning approaches disrupts neural efficiency of the brain compared with implicit approaches [57]. While this explicit framework of instruction is routine, it was proposed recently that a shift to more implicit (less explicit) training strategies might be beneficial, particularly in the initial stages of learning [58].

**Figure 3:**
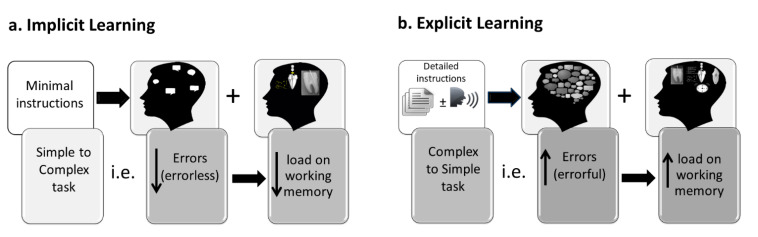
Application of implicit and explicit approaches. (**a**). Implicit learning, (**b**). Explicit learning.

In a study using the Delphi technique to explore opinions by experts regarding descriptions for implicit and explicit learning methods, there was a lack of agreement among experts regarding the application of explicit and implicit motor skill learning [59]. However, there was agreement that certain methods can promote more implicit or more explicit motor learning depending on instructions, limitations in the environment, type of motor task, and personal abilities. Various implicit and explicit motor skill learning strategies have been reported in the literature [48,59]. Methods for learning more implicitly include learning motor skills under secondary task conditions [13] learning without errors [13,60], learning with physical ‘guidance’ [61] [e.g. suture and knot tying; 61], learning by analogy [55] [e.g. table tennis; 55]), or learning from observation [62] [e.g. suture and knot tying. 62]). The efficacy of these various approaches for learning motor skills has been demonstrated in dentistry. For example, in restorative dentistry, using a procedure to reduce the production of errors (i.e., errorless learning: learning from simple to complex task), has been shown to result in significantly higher levels of performance during both learning and testing phases compared with learning explicitly via increasing errors (errorful: from complex to simple task) [6,13]. Errorless (implicit) learning is thought to result in motor skill performance that does not require working memory resources. In contrast, deterioration of performance in the errorful (explicit) learning might be related to an extra load on working memory resources that was needed for completing the dental procedure (Figure 3). Faculty involved in teaching and learning of dental students should be aware of this finding to improve students’ learning as this would impact on their readiness to practice independently in their future career.

Implicit learning tends to minimize hypothesis testing by participants [63]. Indeed, a core principle of implicit learning approaches is to develop learning protocols that minimize hypothesis testing and prevent participants learning from their errors [64]. Despite the increasing body of evidence supporting implicit approaches of motor task learning, most of these studies were related to simple gross motor learning (e.g., sports) [7,57,64] compared to a limited number of studies addressing complex fine motor tasks used in medicine and dentistry [6,13].

In general, motor skill learning strategies that result in high conscious awareness about how the motor task is articulated are used to promote motor learning that is more explicit; for example, by learning motor skills by increasing errors (errorful), trial-and-error learning, and learning by observation combined with instruction [59].

## 4. Conclusions

There is limited evidence regarding fine motor skill learning in dentistry, particularly in endodontics. It has been shown that learning implicitly when carrying out a motor skill may limit the effect of self-focus and self-regulation on subsequent performance.

Stressors related to dental students’ transferring from simulation to clinical settings can result in deterioration of performance. It is suggested that learning implicitly in the simulation stage can reduce disruptions in performance when moving to clinical settings. Therefore, further investigation of effective methods for learning fine dental motor skills is indicated, using conditions that result in robust performance, even under multi-tasking or stressful conditions.

## Figures and Tables

**Table 1:** Description of terms used in this article.

Term	Description
Augmented (extrinsic) feedback	Supplementary or reinforcing feedback received from the surrounding environment related to the movement outcome and the quality of the executed movement.
Block practice	Performing a motor task in a repetitive manner without variation in the practice (e.g., AAA, BBB, CCC).
Chunking	Dividing large pieces of information into smaller elements that are easier to store in the short-term memory.
Cognitive knowledge	Acquiring factual existing information and discovering new knowledge through human thinking.
Declarative knowledge	Descriptive information stored in memory that is static in nature which describes things, events, or processes.
Errorful learning	Learning by loading the learning environment (e.g., instructions, skill difficulty) aiming to increase errors.
Errorless learning	Learning by constraining the learning environment (e.g., instructions, skill difficulty) aiming to reduce errors.
Explicit learning	Learning which generates verbal knowledge of movement performance (i.e., facts and rules) that is dependent on working memory.
External focus	Occurs when the learner’s focus of attention is directed toward the effect of the motor task (e.g., final shape of the cavity preparation).
Extraneous cognitive load	Dependent on how movement information is presented to learner and controlled by the design of instructions.
Generalized motor program	Stored muscle general rules that may be applied to different environmental or situational contexts.
Germane cognitive load	The work put into processing, construction, and automation of movement knowledge to create a permanent store in memory.
Hypothesis testing	Learning by repetitive attempt to perform a task by detecting and correcting errors.
Implicit learning	Learning with minimal increase in verbal knowledge (i.e., facts and rules) of movement resulting in skills that are unconsciously retrieved from memory.
Inherent (intrinsic) feedback	Feedback related to information about motor task gained through sensory channels during or after the execution of the movement.
Internal focus	Occurs when the learner’s focus of attention is directed toward the action of the motor task (e.g., hand movement or bur angulation).
Intrinsic cognitive load	Directly related to learning task and defined by the number and interaction of the processed elements.
Random practice	Performing a motor task in a random manner with variation in the practice (e.g., ABC, BCA, CAB).
Sensory memory	Type of short-term memory that is able to process and recall information related to sensory input.
Working memory	Short-term memory that can store small amount of information for the execution of cognitive processes.

**Table 2:** Summary of motor learning theories relevant to dentistry.

Theory	Description	Points in Favor	Points Against
1. Schema Theory [16]	Motor learning involves ongoing processes that update the recall and recognition of proprioceptive information from limbs and fingers. The response parameters (e.g., speed and force) are specified according to stored knowledge of the results.	- Commonly used theory. - Used to explain procedural skill development.	- No longer valid for understanding motor skill learning. - Unable to describe learning through observation. - Unable to explain the role of augmented feedback.
2. The OPTIMAL theory of motor learning [17]	Focuses on discovering the correct instructional approach to support motivation and direction of motor learning to the desired outcome of the motor task.	- Supports simplifying movement instructions. - Positive impact on instructional design. - Reduces the load on the working memory.	- Limited evidence addressing complex fine motor skill learning.
3. Cognitive Load Theory [18]	Based on the assumption that cognitive system is limited as working memory can only store and process a small amount of information for a few seconds.	- Knowledge build up by combining simple elements can result in development of more complex results. - Reduces the load on working memory. - Positive impact on instructional design.	- Lacks conceptual clarity and validity of instrument used. - Lack generalizability in different contexts. - Uses self-reported survey to measure cognitive loads.
4. Novice-Expert continuum and deliberate practice principles [19]	Development of expert motor performance depends on continuous deliberate practice improved by trial-and-error learning and supported by appropriate supervision.	- Supports gradual buildup and improvement of motor skills. - Supports safe and low risk buildup of competency.	- Does not address individual’s cognitive, attentional and perceptual abilities. - Requires a long deliberate practice to achieve expert level.
5. Reinvestment Theory [20]	Based on the distinction between individual’s movement self-consciousness features related to movement processing and decision making.	- Commonly used theory. - Implicit learning reduces the load on the working memory. - Supports simplifying movement instructions. - Implicit learning maintains robust performance under multi-tasking and stressful conditions.	- Limited evidence addressing complex fine motor skill learning. - Lack of consensus related to the role of observational learning as an implicit learning approach. - Uses self-reported survey to measure the level of reinvestment.
